# Size, Composition, and Phase-Tunable Plasmonic Extinction in Au–Sn Alloy Nanoparticles

**DOI:** 10.1021/acs.jpcc.5c00563

**Published:** 2025-06-09

**Authors:** Connor S. Sullivan, Noah L. Mason, Anthony J. Branco, Sangmin Jeong, Oluwatosin O. Badru, Michael B. Ross

**Affiliations:** Department of Chemistry, 14710 University of Massachusetts Lowell, Lowell, Massachusetts 01854, United States

## Abstract

The synthesis of Au–Sn nanoparticles with tailorable localized surface plasmon resonances (LSPR) is explored, with a focus on size dependence, composition, and phase formation. Au–Sn nanoparticles were synthesized starting from Au seeds ranging in diameter from 5 to 30 nm. UV–visible spectroscopy revealed controllable blueshifting of the LSPR, from 520 to 460 nm, as Sn incorporation increased. X-ray diffraction (XRD) confirmed the formation of Au_5_Sn and AuSn intermetallic phases, with intermetallic formation dependent on both nanoparticle size and Sn content. Elemental analysis through energy-dispersive X-ray spectroscopy (EDS), total reflectance X-ray fluorescence (TXRF), and inductively coupled plasma optical emission spectroscopy (ICP-OES) provided further insight into the incorporation of Sn into Au nanoparticle seeds. We show that this approach allows one to create Au–Sn alloy nanoparticles of varying radii and crystalline phase contents all with the same LSPR (500 nm). Additionally, the size-dependent formation of intermetallic phases provides new physical insight into their impact on the LSPR. Formation of Au_*x*_Sn_1–*x*_ is associated with minimal blueshifting and broadening and Au_5_Sn is associated with linear blueshifting and a small amount of broadening, while AuSn formation leads to rapid blueshifting, broadening, and plasmon damping. This understanding enables precise control over the size, structure, and optical properties of Au–Sn nanoparticles, paving the way for the design of new plasmonic materials for applications in sensing, imaging, and catalysis.

## Graphical abstract

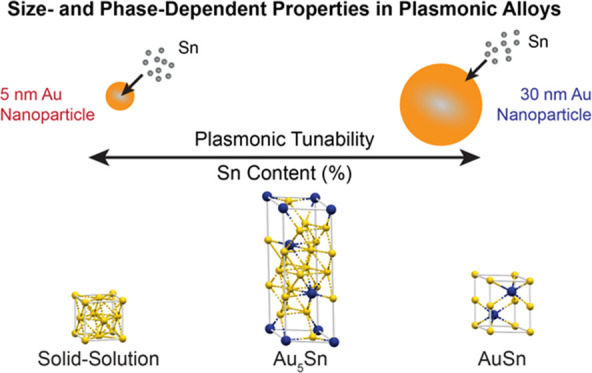

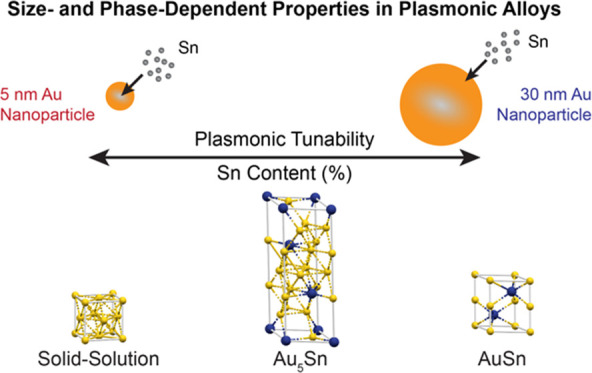

## Introduction

Multimetallic nanoparticles are of interest due to their tunable optical, electronic, and catalytic properties that are strongly influenced by their size, shape, and composition.^1–7^ Among these, Au nanoparticles are well-known for their localized surface plasmon resonance (LSPR), a phenomenon that occurs when conduction band electrons oscillate collectively in response to incident light resulting in efficient absorption and scattering.^8–13^ However, Au nanoparticles are optically limited to visible and infrared LSPRs and are chemically limited due to their relative inertness. In order to expand their range of accessible physical and chemical properties, alloying with other metals, such as post-transition metals, can provide tunable LSPRs and surface chemistry.^3,14^

Post-transition metals offer intriguing properties that can complement those of Au when alloyed or co-deposited; they have ultraviolet plasma frequencies, support UV LSPRs, and can catalyze a range of small molecule reactions.^3,4,15–20^ Post-transition metals also offer distinct chemical reactivity, having shown the ability to activate N_2_, oxidize alcohols, and selectively reduce CO_2_.^20–28^ Recent studies have shown that the synthesis of bimetallic nanoparticles, specifically Au–Sn alloys, can enable the creation of metallic alloys with tunable plasmonic properties that allow for a wider spectral range of light absorption.^3,15,29,30^ The ability to precisely tune the LSPR of these nanoparticles by modifying their composition with respect to nanoparticle size provides a powerful tool for designing next-generation plasmonic materials.^8,31–34^ Of the potential bimetallic combinations, Au–Sn is of interest because of its unique absorption and phase properties, viability as a CO_2_RR catalyst, and its LSPR that can be shifted toward the blue as a function of composition.^26,27,30^ Au–Sn alloy nanoparticles were also found to have measured extinction coefficients within an order of magnitude of pure Au nanoparticles with similar radii while Ag–Sn nanoparticles can absorb in the ultraviolet.^3^ While prior work has enabled creation of phase-pure Au–Sn intermetallic nanoparticles,^35–37^ as well as mixed phase Au–Sn nanoparticles,^3^ none have directly identified the codependence of LSPR location and quality, nanoparticle size, and Sn content.

Here, we present a detailed study of the synthesis of 5, 10, 15, 20, and 30 nm Au–Sn nanoparticles and the systematic tuning of their LSPR by varying the Sn content for each size. By finely controlling the incorporated Sn content, the LSPR can be tuned from 520 to 460 nm. As the total Sn content increases, we show that the nucleation of intermetallic Au_5_Sn and AuSn changes depending on the nanoparticle diameter. This control enables the rational design of plasmon-active nanoparticles at a desired wavelength through different compositions, crystalline morphologies, and Sn content. These findings provide key insight into the size-dependent composition and optical properties of nanoparticle alloys and advance the ability to tailor the desired nanoparticle properties.

## Materials and Methods

### Materials

The synthesis of metal nanoparticles requires Au nanoparticle seed colloids (0.05 mg/mL, Ted Pella), tin­(IV) chloride (SnCl_4_·5H_2_O, 99.99%, Alfa Aesar), poly­(vinylpyrrolidone) (PVP, MW = 40,000, Alfa Aesar), and sodium borohydride (97+%, Alfa Aesar). All chemicals were used without further purification and all solutions were prepared with 18.2 MΩ resistivity water. Syntheses were noted to vary slightly depending on the age of metal salt precursors, most likely due to hydration and oxidation with time.

### Synthesis of Au–Sn Nanoparticles

Au–Sn nanoparticles were synthesized using a seeded approach.^27^ Au colloids of various diameters (5, 10, 15, 20, and 30 nm) at 0.05 mg Au/mL were used. For all sizes, 2.32 mL of the desired diameter Au colloid was added to a 20 mL glass scintillation vial with a stir bar. Next, an amount of distilled water was added based on the amount of Sn that would be added, such that the final volume of the colloid mixture would be 4 mL. Under vigorous stirring, a 10 wt % polyvinylpyrrolidone (PVP) solution was added at a fixed ratio of 20:1 total moles of metal (Au + Sn) to PVP for each synthesis. Next, a 5 mM solution of SnCl_4_ in H_2_O was added, with the volume depending on the desired Sn content. After this, reaction vials were preheated in a 60 °C water bath for 10 min. Then, the colloid mixtures were removed and placed back under vigorous stirring. A fresh solution of 260 mM NaBH_4_ was prepared and rapidly injected into the reaction solution at a fixed ratio of 30:1 moles of NaBH_4_ to total moles of metal (Au + Sn) for each reaction. After stirring for 30 s, the vials were placed back into the 60 °C water bath for 20 min, after which they were removed and allowed to cool to room temperature. To remove excess surfactant and Sn, the nanoparticles were centrifuged, the supernatant was removed, and the pellet was resuspended in water.

### UV–Visible Spectroscopy and Linewidth Analysis

UV–visible spectra were recorded with an Agilent Cary 100 spectrophotometer. A dual-beam setup was employed by using a 1 cm path length quartz cuvette. Dark spectra were acquired to correct for detector noise, and water background spectra were used for background correction. Maxima were identified by the location of the LSPR. Linewidths were determined by quantifying the full width at half-maximum. For Au, these are most accurately determined by doubling the half-width at half-maximum on the low-energy side of the LSPR, to avoid distortion of the lineshape by the interband transitions.

### Powder X-ray Diffraction and Phase Analysis

Powder X-ray diffraction (XRD) measurements were performed on a Rigaku Miniflex X-ray diffractometer using Cu Kα (λ = 1.5418 Å) radiation in the 2θ range of 10–90°, and a scan rate of 1° min^–1^. Samples were prepared by centrifuging 8 mL of colloidal sample, followed by removal of supernatant and resuspension in water. Centrifugation times and speeds varied depending on the Au seed diameter, with 5 nm colloids requiring 1 h at 20,000 r.c.f. to remove from suspension while 30 nm colloids needing 4,500 r.c.f. for 10 min. Samples were then centrifuged again, concentrated to ∼200 μL, drop-cast onto a zero-background Si sample holder (Rigaku), and dried at room temperature. Individual crystal phases were indexed using the crystallographic open-source database. Specific material reference numbers include Au (9013036), Au_5_Sn (1510571), and AuSn (1510301).

### Transmission Electron Microscopy

Transmission electron microscopy was performed using a Phillips CM-12 and HR-TEM/EDS was performed using a JEOL JEM-2100Plus TEM/STEM electron microscope. All imaging was performed at 200 kV. Samples were prepared by drop-casting approximately 5 μL of the washed product onto Cu 200 mesh lacey carbon grids (Ted Pella).

### Total Reflectance X-ray Fluorescence

Total reflectance X-ray fluorescence was performed using a Bruker S2 Picofox instrument with a molybdenum excitation source. Samples were prepared by drop-casting 10 μL of washed product onto a clean quartz disc and then allowed to dry for 1 h. Survey spectra were collected from 0 to 17.5 keV using a standardless method for 1000 s each. The relative composition of Au and Sn was quantified using K_α_ and L_α_ lines by the S2 PICOFOX Control software.

### Inductively Coupled Plasma Optical Emission Spectroscopy

Each measurement was performed on an Agilent 5110 ICP-OES system with Agilent’s ICP Expert software package. Each sample was centrifuged and redispersed in water to remove excess reactants, after which the aliquots were allowed to fully dry in a vacuum oven and were digested with 200 μL of aqua regia. Once fully digested, sample subsets were diluted to 5 mL, and triplicates of ICP-OES were measured. Samples and blanks were compared to calibration curves using a multielement ICP standard.

### Wide-Angle X-ray Scattering

Wide-angle X-ray scattering (WAXS) was performed by using a wavelength of 0.24152 Å on beamline 28-ID-1 at the National Synchrotron Light Source II (NSLS-II) at Brookhaven National Laboratory. All samples were purified by centrifugation and then placed into a Kapton tube. Each Kapton tube was sealed with an epoxy. For background removal, diffraction data of the empty Kapton support and ultrapure water were collected at the same exposure times.

## Results and Discussion

To investigate the size-dependent properties of Au–Sn nanoparticles, we used a seeded synthetic approach (methods, Figure 1). Five different-sized seeds of diameter 5, 10, 15, 20, and 30 nm were chosen. The synthetic approach was adapted from our previously reported work investigating Sn reduction into 13 nm Au nanoparticles.^3^ Transmission electron microscopy (TEM) reveals that the Au–Sn nanoparticles are spherical and relatively monodisperse, with coefficients of variation below 10% for 10–30 nm nanoparticles (Figures S1–S5). As Sn content increases, the nanoparticle diameter increases by 1–2 nm, depending on the starting size (Table S1).

**Figure 1:**
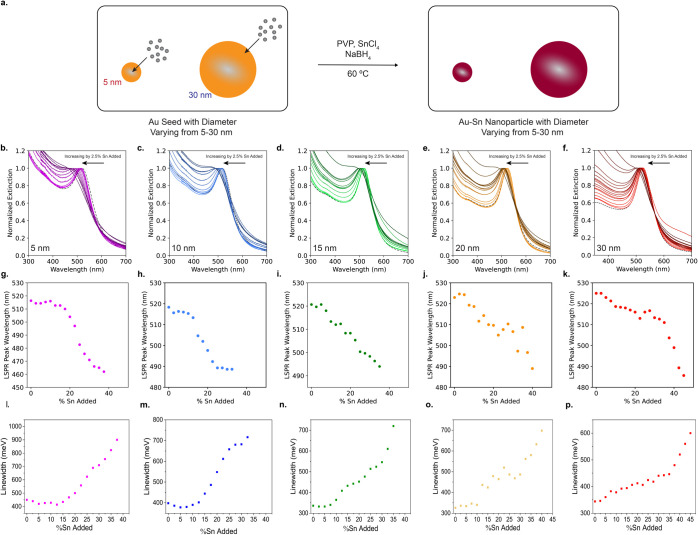
Au–Sn nanoparticle synthesis and optical analysis of synthesized nanoparticles. (a) Scheme showing the synthesis of the Au–Sn nanoparticles. UV–visible spectra of Au–Sn nanoparticles with increasing amounts of Sn added, (b) 5 nm, (c) 10 nm, (d) 15 nm, (e) 20 nm, and (f) 30 nm nanoparticles. LSPR maximum for each Sn-added amount for (g) 5 nm, (h) 10 nm, (i) 15 nm, (j) 20 nm, and (k) 30 nm nanoparticles. LSPR linewidths for increasing amounts of Sn added for (l) 5 nm, (m) 10 nm, (n) 15 nm, (o) 20 nm, and (p) 30 nm nanoparticles.

Figure 1 shows the UV–visible spectra of the five different-sized nanoparticles with the amount of Sn added increased in increments of 2.5% relative to the amount of Au. In all cases, the LSPR blueshifts with increasing Sn amounts added. Given that a few-nm increase in diameter would lead to redshifting of the LSPR, this is due to changes in the electronic properties of the nanoparticle due to alloying.^1,3,4^ The largest LSPR peak shift is seen in the case of 5 nm Au–Sn nanoparticles. In Figure 1, the LSPR of the 5 nm Au–Sn nanoparticles blueshifts from 520 to 460 nm. This is 30 nm greater than the smallest blueshift observed, for the 15 nm Au–Sn nanoparticles. As can be seen in Figure 1g–k, plotting the LSPR location as a function of Sn content shows how LSPR changes differently as a function of the seed size. For small nanoparticle diameters (5 and 10 nm), increases in added Sn amount result in a sigmoidal profile where the LSPR shifts in few-nm increments before rapidly shifting over a relatively narrow Sn content range before stabilizing. For the larger sizes (15, 20, and 30 nm), a relatively linear trend is observed as the added Sn content amount increases. For the two largest diameter 20 and 30 nm nanoparticles, deviation from a linear trend begins following ∼25% Sn addition, resulting in a more rapid blueshift before losing the LSPR lineshape (Figure 1j,k).

In all cases, the lineshape of each resonance broadens until no LSPR is visible as the Sn content increases. Prior work suggests this is due primarily to damping mechanisms ascribed to either electronic changes in the alloy or poor intrinsic plasmonic properties of the intermetallic phases.^3^ To understand this quantitatively, we measured the linewidth of each LSPR by fitting the full width at half-maximum (fwhm) for each spectrum (Figure 1l–p). We find that linewidth increases as a function of Sn content for all nanoparticle sizes. Notably, the trend in linewidth is similar to that in the LSPR location, suggesting a common physical mechanism. Specifically, a sigmoidal trend is observed for the 5 and 10 nm nanoparticles (Figure 1l,m), and a relatively linear trend is observed for the 15, 20, and 30 nm nanoparticles (Figure 1n–p). Notably, the linewidths broaden rapidly at the highest Sn amounts added for the 20 and 30 nm diameter nanoparticles.

To understand the size-dependent optical properties observed in Figure 1, powder X-ray diffraction (XRD) was used to characterize the alloys and intermetallic phases formed as the amount of Sn increases. As the amount of Sn added increases, the phases present in the nanoparticles change (Figure 2). Specifically, it is observed that at low Sn content, only Au fcc reflections are seen, implying any Sn incorporation is in the form of a solid-solution Au_*x*_Sn_1–*x*_ alloy. With increasing Sn content, a reflection at 39.2°—characteristic of Au_5_Sn—is observed. As the seed size increases, the Au_5_Sn intermetallic forms with smaller amounts of Sn added. Based on the XRD data, it is observed that the Au_5_Sn alloy forms at 27.5% Sn added for the 5 nm nanoparticles. The Sn content needed for visible Au_5_Sn in XRD decreases as the seed size increases, implying that smaller-sized Au–Sn nanoparticles require more Sn added to nucleate the more complex intermetallic phases.^16^ This suggests that the optical property differences between small and large diameter nanoparticles can be understood by phase analysis, which is further supported by imaging that reveals the coexistence of the different phases within a particle for this synthesis.^38^ For small diameter (5 and 10 nm) Au–Sn nanoparticles, the lack of a significant LSPR shift at low Sn-added concentrations corresponds to a lack of intermetallic formation.

**Figure 2:**
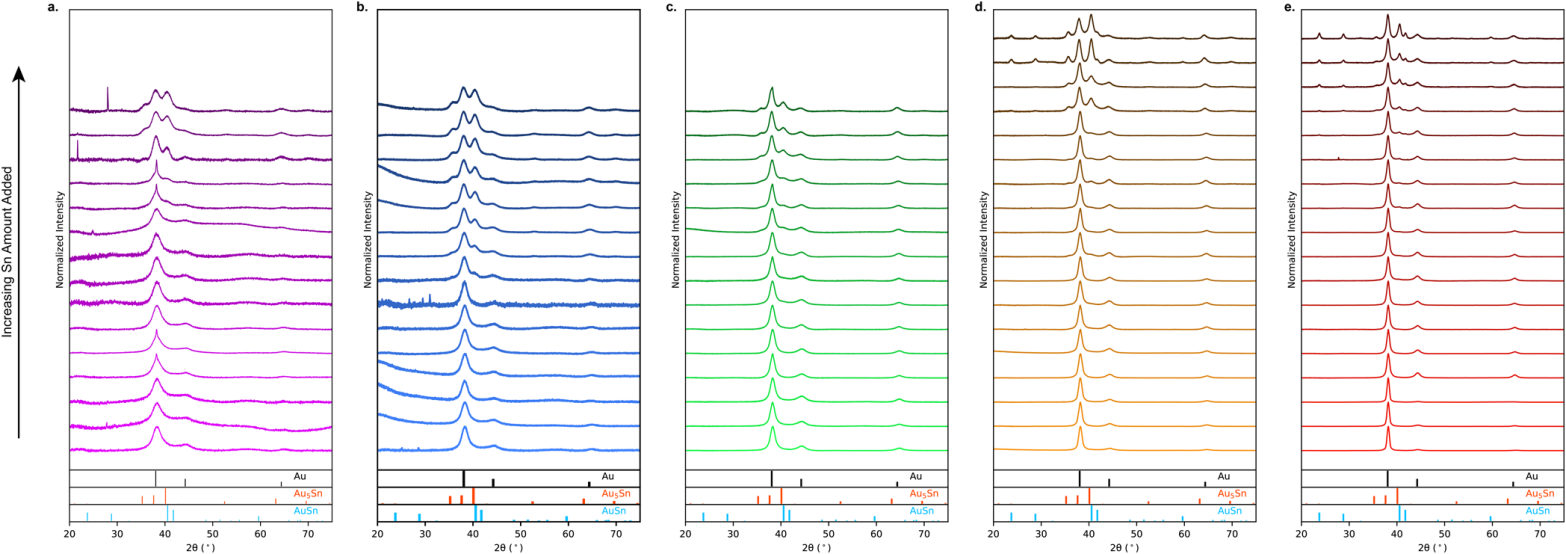
XRD of Au–Sn nanoparticles with increased amounts of Sn added. Increasing Sn-added amounts in 2.5% increments, relative to the Au content, for (a) 5, (b) 10, (c) 15, (d) 20, and (e) 30 nm nanoparticle seeds.

The more Sn-rich phase AuSn is not observed in the 5, 10, and 15 nm XRD for any of the amounts added here, where LSPRs are still observable (Figure 2c–e). In contrast, for the 20 and 30 nm Au nanoparticle seeds, characteristic peaks at 23.2° and 28.1° for the AuSn intermetallic are seen. In the 20 nm nanoparticles, the two AuSn intermetallic peaks form when 37.5% Sn relative to Au is added. These same peaks are seen with less Sn added, 27.5%, in the 30 nm nanoparticles. This is an analogous trend observed for Au_5_Sn phase formation, where larger nanoparticles are more capable of forming Sn-rich intermetallic phases. The presence of the AuSn intermetallic phase also corresponds to the sharp blueshift observed in the extinction of 20 and 30 nm nanoparticles after ∼25% Sn addition (Figure 1o,p), while AuSn is only observed at the highest Sn amounts added for the 15 nm nanoparticle seeds. The latter coincides with the area where significant broadening is observed (Figure 1n). Solution wide-angle X-ray scattering (WAXS) of each sized nanoparticle alloy further confirms this phase behavior and the lack of Sn-rich phases in smaller 5 and 10 nm nanoparticles up to 50% Sn added (Figure S6).

To better understand how the amount of Sn affects the alloying of the nanoparticles at each size, energy-dispersive X-ray spectroscopy (EDS) and total reflectance X-ray fluorescence (T-XRF) were used to quantify the Au and Sn content. In all cases, increasing the Sn added increases the Sn amount incorporated in the final nanoparticle alloy (Tables S2–S6). TXRF values were found to be consistent both in trend and in Sn amount with inductively coupled plasma optical emission spectroscopy (ICP-OES), Tables S2–S6. In Figure 3, the amount of Sn incorporated at the point at which a given intermetallic phase is observed is shown. Confirmed by TXRF, the Au_5_Sn phase begins to form at 6.19% Sn incorporated in the 10 nm nanoparticles, while 5 nm nanoparticles require 15.56% Sn incorporated to develop Au_5_Sn. Notably, the AuSn intermetallic phase begins to form around 9% for 20 nm nanoparticles and requires less for 30 nm nanoparticles. These data further support the previous optical and phase behavior trends, with the smallest 5 nm Au–Sn nanoparticles capable of incorporating significant fractions of Sn before nucleation of the Au_5_Sn intermetallic phase.

**Figure 3:**
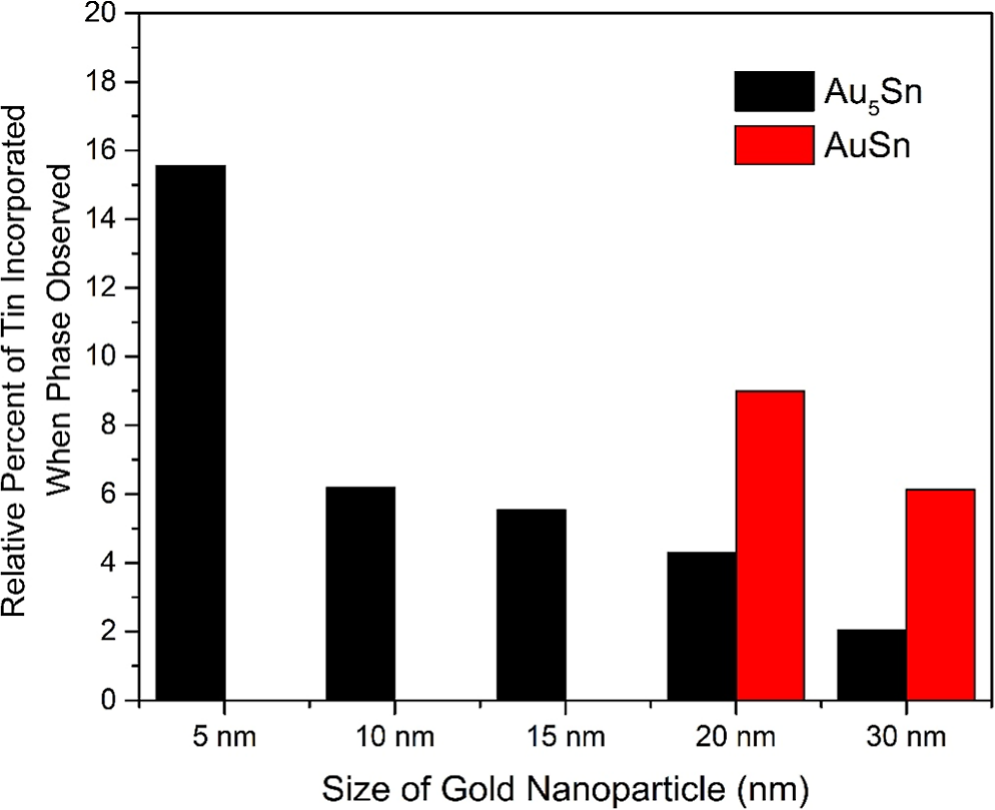
Phase nucleation changes as a function of Sn incorporation into Au–Sn nanoparticles. Plot showing the amount of Sn incorporated when the two intermetallic phases are observed for 5, 10, 15, 20, and 20 nm nanoparticles.

After understanding the physical and phase properties of these Au–Sn nanoparticles, we sought to design nanoparticles of different sizes and compositions that have an equivalent LSPR maximum. For example, an LSPR maximum absorption of 500 nm, not attainable with pure Au nanoparticles, can be achieved in five different ways, with nanoparticles with distinct compositions and crystalline phase structures (Figure 4a, Tables 1 and S7). For the smallest 5 nm nanoparticles, XRD shows only fcc Au reflections, indicating that the changes in absorption are achieved by Au_*x*_Sn_1–*x*_ solid-solution alloy formation. For 15 nm, more Sn is incorporated, leading to the formation of the Au_5_Sn intermetallic to achieve 500 nm absorption (Figure 4b). For the largest nanoparticles, all three phases (Au_*x*_Sn_1–*x*_, Au_5_Sn, and AuSn) are necessary. This suggests that size-dependent changes in LSPR tunability are due to a complex combination of the Sn content and phase.

**Figure 4:**
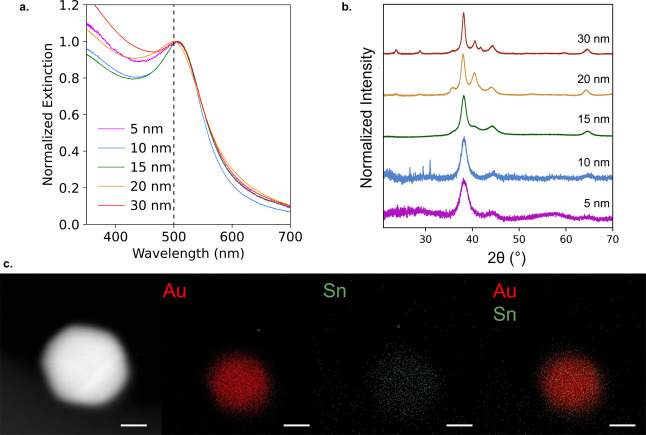
Designed absorption of Au–Sn nanoparticles at 500 nm. (a) UV–visible spectrum of five nanoparticles with designed LSPRs at 500 nm. The dashed line highlights the LSPR for pure Au seeds at ∼520 nm. (b) XRD of five nanoparticles with designed LSPRs at 500 nm. (c) STEM image and EDS maps of a 30 nm Au–Sn nanoparticle with 40.0% Sn added. All scale bars are 10 nm.

**Table 1:** Percent Added and Incorporated Sn for Five Au–Sn Nanoparticles with a Designed LSPR at 500 nm Obtained by TXRF

nanoparticle diameter (nm)	Sn added (%)	Sn incorporated (%)	crystalline phases observed
5 nm	22.5	7.52	Au
10 nm	17.5	4.24	Au
15 nm	25.0	7.60	Au, Au_5_Sn
20 nm	37.5	7.88	Au, Au_5_Sn, AuSn
30 nm	40.0	6.14	Au, Au_5_Sn, AuSn

TEM-EDS maps of the Au–Sn nanoparticles with a designed LSPR of 500 nm show that Sn is present in each nanoparticle for all diameters (Figure S7). This is most clearly observed in the 30 nm nanoparticle, where the highest resolution map was acquired; here, Sn is seen to be uniformly incorporated into the Au nanoparticle seed (Figure 4c).

## Conclusion

This study demonstrates the successful synthetic, compositional, and structural tuning of Au–Sn nanoparticles, highlighting the influence of seed size and Sn content on their plasmonic properties. By employing a seeded synthetic approach, we have shown that varying the Au-to-Sn ratio and the size of the Au seed allows for precise control over the nanoparticle composition and optical properties, enabling tailored blueshifts of the LSPR. Our results indicate that as the seed size increases, the amount of Sn required for the formation of the Au_5_Sn alloy decreases, thus providing a new strategy for tuning the alloy phase of these nanoparticles. Additionally, the formation of an AuSn intermetallic phase was linked to significant blueshifting and broadening of the LSPR, further supporting the critical role of structural factors in dictating the plasmonic behavior of these materials. These clarify our prior results, which indirectly found impacts of Au_5_Sn and AuSn intermetallic formation on LSPR location.^3^ Here, the primary physical insight is that we directly observed that the presence of Au_*x*_Sn_1–*x*_ solid solution drives some blueshifting of the LSPR, while the presence of Au_5_Sn is associated with greater linear blueshifting. The most Sn-rich phase, AuSn, correlates with a significant LSPR shift but also coincides with significant damping and broadening. In contrast with our previous work—which only focused on one nanoparticle diameter—directly controlling nanoparticle size enabled better deconvolution of phase formation from Sn content and its physical effect on LSPR location and broadening.

The tunability of this method was shown to be precise, and increments of 2.5% Sn added to the synthesis can reliably tune the LSPR of the Au–Sn nanoparticles. The ability to finely control the LSPR, ranging from 520 to 460 nm, and achieve a designed LSPR peak at 500 nm across different particle sizes underscores the versatility of Au–Sn nanoparticles for applications in areas such as sensing, imaging, and catalysis.^21,22,26–28^ Annealing in situ could give direct insight into the phase reorganization and transition in these systems, helping to understand both the LSPR location and linewidth dependence on the phase.

This work is important for the development of CO_2_ reduction catalysts, given that both Au and Sn have well-known activity toward this reaction. Prior work on Au–Cu solid solution and intermetallics shows that fine control over the alloy structure can change catalytic activity due to changes in the active site.^39^ Work on Au–Sn alloys has shown that Sn content changes the activity, with the AuSn intermetallic phase supporting the highest rates.^27^ Within the context of this work, Sn alloying impacts both the LSPR and the atomic arrangement, which together can impact both charge transfer and molecular adsorption, enabling fine control over catalysis. These findings provide valuable insights into the design of next-generation plasmonic materials, advancing the potential for tailored optical responses for a range of technological applications.

## Supplementary Material



## Notes

Size and compositional data for all nanoparticles investigated, additional electron microscopy, and X-ray scattering data (PDF)
