# Supplementary material for: Size, Composition, and Phase-Tunable Plasmonic Extinction in Au–Sn Alloy Nanoparticles
Source: J Phys Chem C Nanomater Interfaces. 2025 Jun 9;129(24):11070–6. doi: 10.1021/acs.jpcc.5c00563 (PMC12186605; doi:10.1021/acs.jpcc.5c00563)
Supplement: Supplementary file 1 [file jp5c00563_si_001.pdf]

Size, Composition, and Phase Tunable Plasmonic Extinction in Au-Sn Alloy Nanoparticles

Connor S. Sullivan,[+] Noah L. Mason,[+] Anthony J. Branco, Sangmin Jeong, Oluwatosin O. Badru, and Michael B. Ross[*]

[1]Department of Chemistry, University of Massachusetts Lowell, Lowell, MA 01854

+These authors contributed equally to this work

*Corresponding Author

Supporting Tables

Table S1. Size distribution of Au-Sn nanoparticles measured using HR-TEM.

5nm	Particle Size (nm)	Coefficient of Variation
10%	5.43 ± 0.62	0.11
20%	5.31 ± 0.72	0.14
30%	6.02 ± 0.98	0.16
40%	5.92 ± 1.01	0.17
50%	6.44 ± 0.86	0.13
10 nm		
10%	10.21 ± 0.15	0.01
20%	10.10 ± 0.42	0.04
30%	10.35 ± 0.25	0.02
40%	10.05 ± 0.31	0.03
50%	10.73 ± 0.67	0.06
15 nm		
10%	15.25 ± 0.09	0.01
20%	15.12 ± 0.12	0.01
30%	15.04 ± 0.48	0.03
40%	15.03 ± 0.36	0.02
50%	15.09 ± 0.38	0.03
20 nm		
10%	20.22 ± 0.14	0.01
20%	20.65 ± 0.23	0.01
30%	20.05 ± 0.18	0.01
40%	20.84 ± 0.44	0.02
50%	21.06 ± 0.96	0.05
30 nm		
10%	30.11 ± 1.56	0.05
20%	30.62 ± 1.23	0.04
30%	29.08 ± 2.03	0.07
40%	31.26 ± 1.55	0.05
50%	31.45 ± 1.28	0.04

Table S2. Sn content of ~5 nm Au-Sn nanoparticles measured by TXRF and ICP-OES.

	10% Sn-Added	20% Sn-Added	30% Sn-Added	40% Sn-Added	50% Sn-Added
TXRF	5.20	5.67	15.56	17.54	23.97
ICP-OES	3.18	4.85	13.67	19.44	27.40

Table S3. Sn content of ~10 nm Au-Sn nanoparticles measured by TXRF and ICP-OES.

	10% Sn-Added	20% Sn-Added	30% Sn-Added	40% Sn-Added	50% Sn-Added
TXRF	1.33	3.19	6.19	14.14	29.34
ICP-OES	1.12	3.05	8.33	19.43	24.72

Table S4. Sn content of ~15 nm Au-Sn nanoparticles measured by TXRF and ICP-OES.

	10% Sn-Added	20% Sn-Added	30% Sn-Added	40% Sn-Added	50% Sn-Added
TXRF	0.99	5.54	13.21	24.53	22.43
ICP-OES	0.33	4.31	10.31	17.92	24.13

Table S5. Sn content of ~20 nm Au-Sn nanoparticles measured by TXRF and ICP-OES.

	10% Sn-Added	20% Sn-Added	30% Sn-Added	40% Sn-Added	50% Sn-Added
TXRF	1.24	4.31	3.98	9.00	14.64
ICP-OES	0.53	2.57	3.22	6.54	14.80

Table S6. Sn content of ~30 nm Au-Sn nanoparticles measured by TXRF and ICP-OES.

	10% Sn-Added	20% Sn-Added	30% Sn-Added	40% Sn-Added	50% Sn-Added
TXRF	2.22	2.05	0.99	6.14	9.64
ICP-OES	1.53	1.51	0.88	4.38	11.45

Table S7. Percent composition of 5 Au-Sn nanoparticles with a designed LSPR at 500 nm measured using EDS.

NP Size	Au (At.%)	Sn (At.%)
5nm	98.53	1.47
10nm	97.69	2.31
15nm	93.96	6.04
20nm	96.84	3.16
30nm	97.71	2.29

Supporting Figures

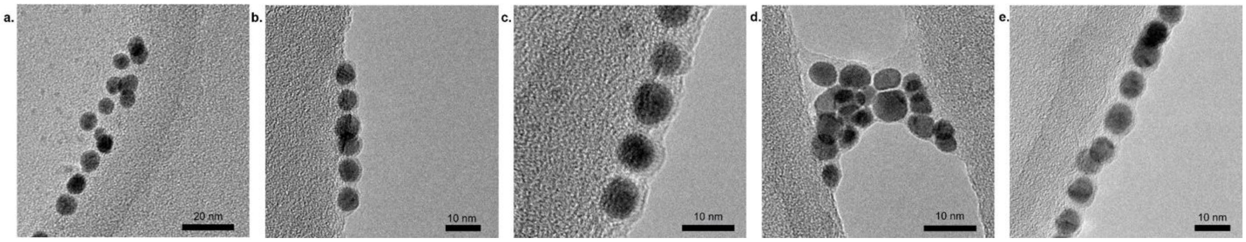

Figure S1. TEM images of 5 nm Au-Sn nanoparticles. (a.) 10% Sn added, (b.) 20% Sn added, (c.) 30% Sn added, (d.) 40% Sn added, and (e.) 50% Sn added.

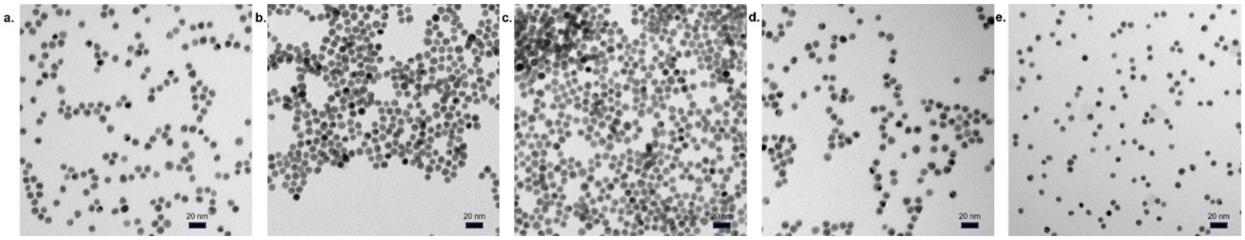

Figure S2. TEM images of 10 nm Au-Sn nanoparticles. (a.) 10% Sn added, (b.) 20% Sn added, (c.) 30% Sn added, (d.) 40% Sn added, and (e.) 50% Sn added.

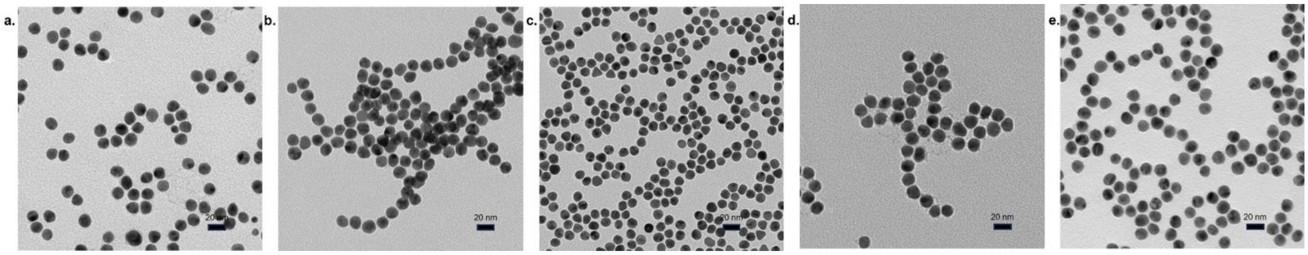

Figure S3. TEM images of 15 nm Au-Sn nanoparticles. (a.) 10% Sn added, (b.) 20% Sn added, (c.) 30% Sn added, (d.) 40% Sn added, and (e.) 50% Sn added.

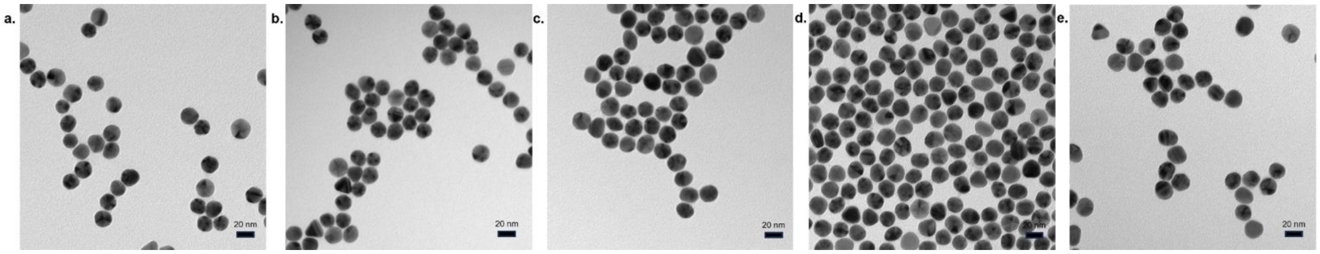

Figure S4. TEM images of 20 nm Au-Sn nanoparticles.(a.) 10% Sn added, (b.) 20% Sn added, (c.) 30% Sn added, (d.) 40% Sn added, and (e.) 50% Sn added.

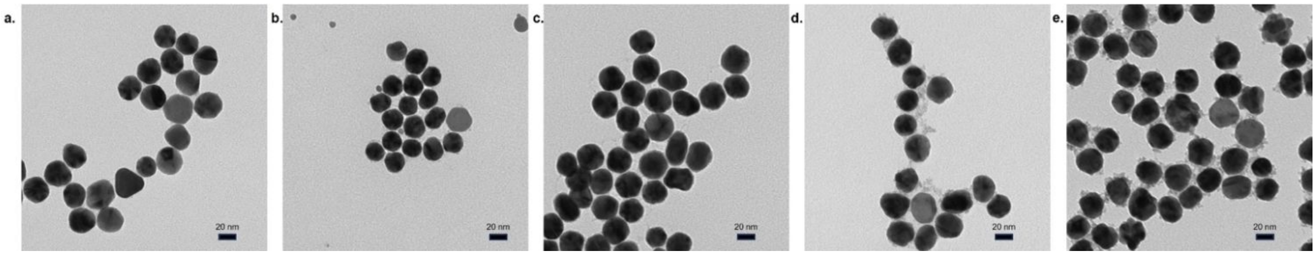

Figure S5. TEM images of 30 nm Au-Sn nanoparticles.(a.) 10% Sn added, (b.) 20% Sn added, (c.) 30% Sn added, (d.) 40% Sn added, and (e.) 50% Sn added.

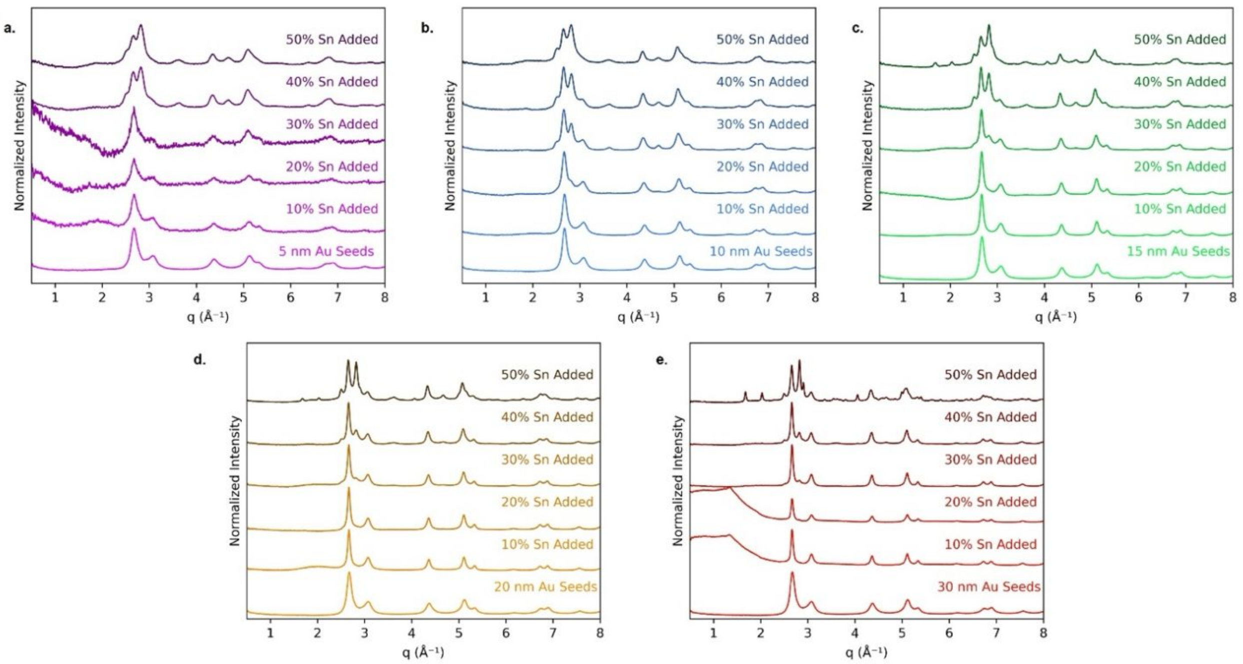

Figure S6. WAXS of Au-Sn nanoparticles with increasing amounts of Sn added. (a.) 5 nm nanoparticles, (b.) 10 nm nanoparticles, (c.) 15 nm nanoparticles, (d.) 20 nm nanoparticles, and (e.) 30 nm nanoparticles.

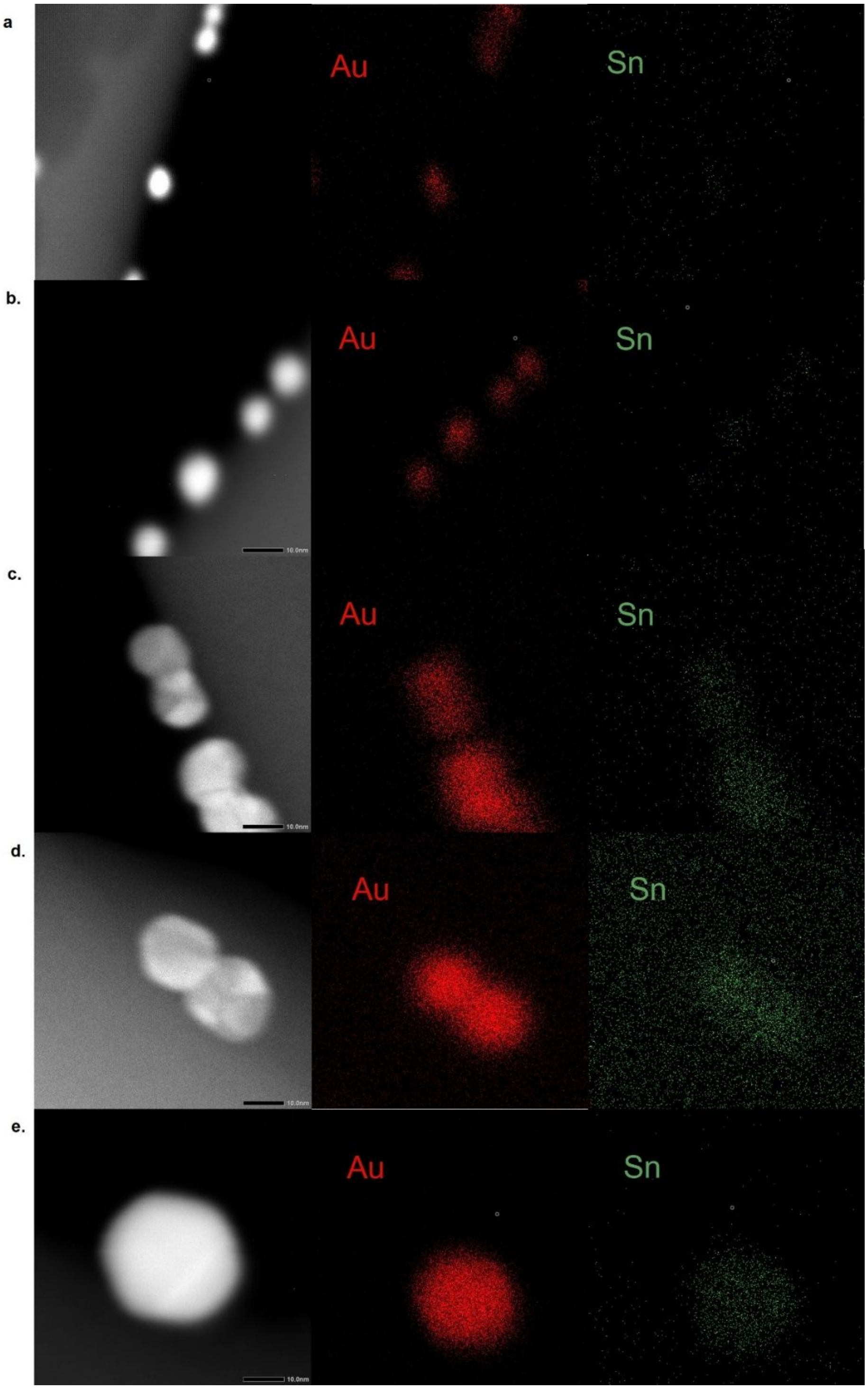

Figure S7. STEM-EDS of five Au-Sn nanoparticles designed with an LSPR at 500nm. (a.) 5 nm nanoparticles, (b.) 10 nm nanoparticles, (c.) 15 nm nanoparticles, (d.) 20 nm nanoparticles, and (e.) 30 nm nanoparticles.
